# Endometrial Carcinomas with Intestinal-Type Metaplasia/Differentiation: Does Mismatch Repair System Defects Matter? Case Report and Systematic Review of the Literature

**DOI:** 10.3390/jcm9082552

**Published:** 2020-08-06

**Authors:** Laura Ardighieri, Andrea Palicelli, Federico Ferrari, Mattia Bugatti, Emma Drera, Enrico Sartori, Franco Odicino

**Affiliations:** 1 Pathology Unit, ASST Spedali Civili di Brescia, Piazzale Spedali Civili 1, 25123 Brescia, Italy; lauraardighieri@gmail.com (L.A.); bgtmtt@hotmail.it (M.B.); emy79na@gmail.com (E.D.); 2 Pathology Unit, Azienda Unità Sanitaria Locale-IRCCS, Viale Risorgimento 80, 42123 Reggio Emilia, Italy; andreapalicelli@hotmail.it; 3 Department of Obstetrics and Gynecology, ASST Spedali Civili di Brescia, Piazzale Spedali Civili 1, 25123 Brescia, Italy; federicogferrari@gmail.com; 4 Department of Molecular and Translational Medicine, School of Medicine, University of Brescia, 25125 Brescia, Italy; 5 Department of Clinical and Experimental Sciences, University of Brescia, Viale Europa 11, 25123 Brescia, Italy; enrico.sartori@unibs.it

**Keywords:** intestinal, metaplasia, mucinous, goblet cells, mismatch repair, endometrial carcinoma

## Abstract

Background: Intestinal metaplasia/differentiation in primary endometrial carcinomas is an uncommon phenomenon, with only few cases described. Material and Methods: We performed a systematic review of endometrial carcinomas with intestinal metaplasia/differentiation interrogating the electronic databases Pubmed, Web of Science, and Scopus, and we reported an additional case arising in a 49-year-old woman. Results: We identified only eight patients diagnosed with endometrial carcinomas exhibiting intestinal metaplasia/differentiation, and additionally our case. Endometrial carcinomas with intestinal-type features can present in pure or mixed forms in association with usual-type endometrioid carcinomas; in mixed forms, the two neoplastic components may derive from a common neoplastic progenitor, as evidenced by the concomitant loss of MSH2 and MSH6 protein expression in our case. Disease recurrences occur in a significant fraction of the cases, including patients diagnosed in low-stage disease. Conclusions: Endometrial carcinomas with intestinal metaplasia/differentiation are rare and they may represent a more aggressive tumor variant, thus requiring a proper treatment despite the low-tumor stage. The ProMise classification should be performed also in these unusual tumors, since they can be associated with mismatch repair system defects.

## 1. Introduction

Endometrioid carcinoma is the most common endometrial cancer, often showing a wide spectrum of morphological variants and metaplastic changes that can make the diagnosis challenging [1,2,3]. Mucinous differentiation in endometrioid carcinomas is a frequent phenomenon; however, according to the criteria first proposed by Ross in 1983 [4] and subsequently adopted by the World Health Organization (WHO) classification [1], only endometrial carcinomas (ECs) composed of >50% by mucinous cells are classified as mucinous carcinomas. Primary mucinous carcinomas of the endometrium comprise 1–9% of all ECs, usually showing an endocervical-type differentiation [5,6]. They are typically well-differentiated with a relatively good prognosis; however, aggressive cases have been reported [1,7].

Rare variants of mucinous differentiation were described in ECs, including gastric-type and intestinal-type [8,9]. Gastric (gastrointestinal)-type differentiation in malignant ECs has been recently elucidated by Wong and Colleagues who proposed diagnostic criteria for its recognition. According to the authors, gastric (gastrointestinal)-type mucinous carcinomas represent a rare and aggressive subtype of EC with specific morphological and immunohistochemical features, including the absence of an endometrioid component [8].

Intestinal type metaplasia/differentiation (IM/diff) in EC was first described by Berger in 1984 [10]. Since then, it has been exceptionally reported in pure forms or in association with endometrioid carcinomas [11]; for its rarity, clinical implications are unclear. We performed a systematic literature review to provide additional clinico-pathological information helpful in understanding this unusual finding and we discussed the spectrum of differential diagnoses (including primary and secondary endometrial tumors with intestinal/intestinal-like features) and possible pathogenic hypotheses. In addition, we reported a new case of EC showing mixed endometrioid and IM/diff, with Mismatch Repair System (MMR) defects.

## 2. Materials and Methods

A systematic literature review was performed according to the PRISMA (Preferred Reporting Items for Systematic Reviews and Meta-Analyses) guidelines. No Institutional Review Board (IRB) approval was required for this study [12].

The study aimed to answer the following PICOS (Population, Intervention, Comparison, Outcomes) questions:

- Population: patients with a diagnosis of endometrial carcinoma with IM/diff;
- Intervention: any type of treatment, including surgery, chemotherapy, radiotherapy, or observational treatment;
- Comparison: no comparisons are expected;
- Outcomes: patient’s status at last follow-up: no evidence of disease (NED), AWD (alive with disease), dead of disease (DOD);
- Study design: observational study (retrospective case series, case reports).

The eligibility/inclusion criteria were studies in English Language and studies describing endometrial carcinomas with IM/diff. Exclusion criteria were cases arising outside the uterine body, cases showing other types of mucinous differentiation (endocervical-type, undifferentiated-type, and gastric-type), and cases with uncertain diagnosis.

Information sources and search strategy: we searched for (endometrium or endometrial or endometrioid or “uterine body” or “uterine corpus”) and (carcinoma or carcinomas or carcinoma OR carcinomas) and (intestinal or goblet or enteric or Paneth) in Pubmed (all fields), Web of Science (Topic/Title) and Scopus (Title/Abstract/Keywords) databases. No limitations or additional filters were set. All relevant articles were obtained in full-text format (see study selection) and screened for additional references. The bibliographic research ended on 6 June 2020.

Study selection: two independent reviewers (L.A. and A.P.) selected the studies using a two-steps screening method. In the first-step, screening of abstracts and titles was performed to verify eligibility/inclusion criteria and to exclude irrelevant studies. In the second step, full texts of all relevant articles were screened by the two reviewers to: (1) verify study eligibility and inclusion criteria and (2) avoid duplications of the included cases. Two other authors (F.F. and E.D.) performed a manual search of reference lists in order to avoid missing of additional relevant or recent publications. E.S. and F.O. checked the data extracted.

Object of the systematic review: to update and summarize the literature concerning carcinomas arising in the endometrium showing IM/diff and to report any information regarding clinical characteristics, tumor pathological features, treatment strategies, and patients’ outcomes.

Data collection process/data items: data collection was study-related (authors and year of study publication) and case-related (patient age, tumor morphological, immunohistochemical and molecular features, neoplastic precursor lesions, disease’s stage at presentation, treatment, and outcomes).

Statistics: for statistical analysis, the collected data were reported as continuous or categorical variables. Categorical variables were summarized by frequency and percentage; continuous variables were summarized by ranges and mean and median values where appropriate. Time-to-recurrence was the time between primary surgery to disease recurrence. The survival status was the time from primary surgery to the last follow-up.

## 3. Results

### 3.1. Case Report

A 49-year-old woman (gravida 2, para 1) presented abnormal uterine bleeding and persistent pelvic pain. Menopause occurred at 47 years of age, and she did not receive any hormonal replacement therapy. Her body mass index was 29. Patient’s history was unremarkable as that of her first-degree family members, while two second-degree relatives were diagnosed with EC. Transabdominal ultrasonography revealed abnormally thickened endometrium (16 mm) with features suggestive for an EC invading >50% of the myometrium. Pre-operative hysteroscopic biopsy demonstrated a moderately differentiated (G2) endometrioid carcinoma of the endometrium. CA125 serum levels were increased (697 mU/L). Pathological iliac pelvic and para-aortic lymph nodes were found on staging thoraco-abdominal computed tomography (CT) scans. Abdominal Magnetic Resonance (MR) confirmed a solid, isointense endometrial tumor invading the deep myometrium and the cervical stroma; in addition, a metastasis with diffusion-weighted Imaging (DWI) restriction signal was detected in the liver. At multidisciplinary evaluation, the patient was diagnosed with FIGO Stage IVB EC (cT1b cN2 cM1). After 8 cycles of palliative Carboplatinum and Paclitaxel-based chemotherapy, treatment response was evaluated. CA125 normalized (28 mU/L); AFP, CEA, HE4, and hCG were negative, while CA19.9 and CA15.3 were altered (both 72 mUl/L). CT and MR scan demonstrated a partial response according to RECIST (Response Evaluation Criteria in Solid Tumours) criteria. She underwent multidisciplinary reassessment, and she was proposed and hence submitted to palliative laparoscopic hysterectomy with bilateral adnexectomy and pelvic lymphadenectomy.

On gross examination, an intrauterine 2 × 1.5 × 1.5 cm, firm, whitish tumor mass centered in the low-uterine segment was detected, invading the outer half of the myometrium. The cervix, ovaries, and fallopian tubes were unremarkable.

**Figure 1:**
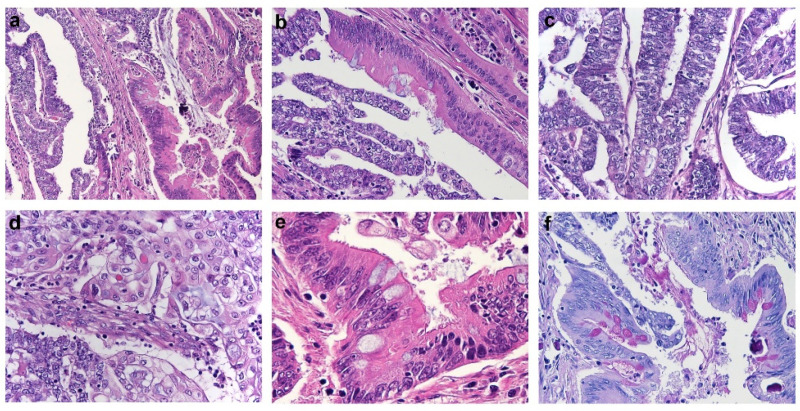
Histological features of Endometrial Carcinoma showing intestinal-type features. (**a**) Endometrial carcinoma showing mixed endometrioid (right half) and intestinal-type mucinous (left half) features (H&E). (**b**) Single tumor gland showing concomitant endometrioid and intestinal-type differentiation (H&E). (**c,d**) Morphological details of the endometrioid component, composed by glands lined by columnar cells with scant cytoplasm (**c**), and foci of squamous differentiation (**d**) (H&E). (**e**) Morphological details of the intestinal-type component composed by enterocyte-like cells with apical brush border and goblet cells (H&E). (**f**) PAS-D istochemical stain highlighting the presence of intracytoplasmic mucin in the goblet cells of the intestinal-type tumor component (PAS-D stain).

Histological examination revealed an EC showing two different tumor components, one with endometrioid features and the remaining with mucinous features in the form of IM/diff (Figure 1a). The percentage of endometrioid and mucinous components was almost equal: in some areas they were separated, while in others they were tightly connected and intermingled each other, occasionally being both identified in the same gland (Figure 1b).

The endometrioid component exhibited predominant glandular architecture; it was mostly represented by columnar cells with grade 2–3 nuclei and scarce cytoplasm (Figure 1c) and showed focal areas of squamous differentiation (Figure 1d) and papillary growth. The mucinous component with IM/diff was predominantly composed of single-layered glands lined by columnar cells with grade 1–2 nuclei and abundant mucinous cytoplasm. In addition, tall enterocyte-like cells with apical brush border and goblet cells were identified in some of the glands (Figure 1e), and PAS diastase stain (PAS-D) istochemical stain highlighted the presence of intracytoplasmic mucin in the goblet cells (Figure 1f). Paneth cells were not found.

Lymphovascular invasion was prominent. A brisk mixed inflammatory infiltrate was detected in tumor stroma and glandular lumens. The tumor deeply invaded the outer half of the myometrium with focal microscopic involvement of the endocervical stroma; the cervix, entirely submitted for histological evaluation, showed no evidence of endocervical-adenocarcinoma precursors (usual-type or gastric-type adenocarcinoma in situ, lobular endocervical glandular hyperplasia, and endometriosis). The ovaries, Fallopian tubes, para-uterine tissues, and thirteen pelvic lymph nodes were free of tumor.

**Figure 2:**
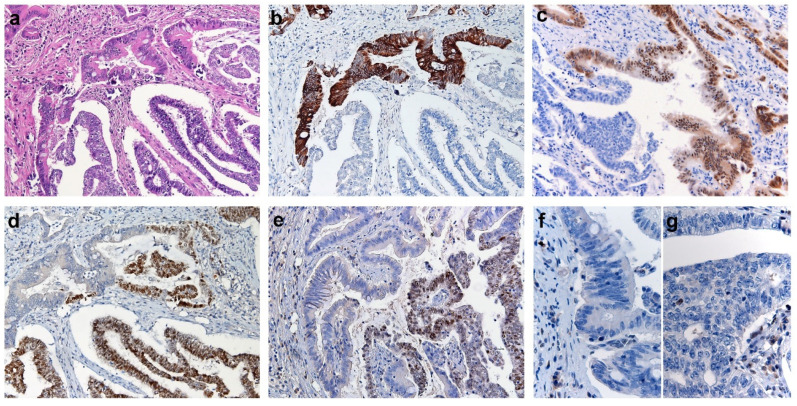
Immunohistochemical features of Endometrial Carcinoma showing intestinal-type features. (**a**) Representative H&E section of the endometrial carcinoma showing two tumor components, the intestinal differentiated one in the upper half and the endometrioid one in the lower half. (**b**–**e**) Immunohistochemical stainings for CK20 (**b**), CDX2 (**c**), PAX8 (**d**) and ER (**e**); the intestinal differentiated component (upper half) is positive for CK20 (**b**) and CDX2 (**c**) while negative for PAX8 (**d**) and (ER); the endometrioid component (lower half) shows opposite profile with positivity for PAX8 (**d**) and ER (**e**) and negativity for CK20 (**b**) and CDX2 (**c**). Immunohistochemical staining for MSH6 showing protein loss of expression in the intestinal (**f**) and endometrioid (**g**) tumor components.

At immunohistochemical analysis, both tumor components were negative for germ cell markers (AFP, SALL4, and Glypican-3), while they showed distinctive expression of Müllerian and intestinal markers (Table 1) (Figure 2).

The endometrioid component (Figure 2a lower half) was negative for CK20 (Figure 2b) and CDX2 (Figure 2c) while diffusely positive for PAX8 (Figure 2d), ER (Figure 2e) and PR; the mucinous areas with IM/diff (Figure 2a upper half) showed opposite profile with diffuse positivity for CK20 (Figure 2b) and CDX2 (Figure 2c) and negativity for PAX8 (Figure 2d), ER (Figure 2e) and PR. Interestingly, investigation of MMR proteins revealed loss of MSH2 and MSH6 (Figure 2g–f) proteins in both tumor components (in the presence of internal positive control) and retained MLH1 and PMS2 expression in both tumor components

In addition, molecular analysis identified high-grade microsatellite instability (MSI-H) in tumor tissue. Background endometrium was atrophic without evidence of IM/diff or atypical endometrial hyperplasia (AEH); however, evaluation of MMR proteins revealed focal loss of MSH2 and MSH6 expression in normal-appearing endometrial glands. A diagnosis of mixed endometrioid and mucinous-intestinal type differentiated EC with MMR defects was made. The patient underwent colonoscopy and esophagogastroduodenoscopy (EGD), which failed to reveal abnormalities in the explored sites. The patient is alive with persistence of the hepatic metastasis 6 months after surgery.

### 3.2. Literature Review

**Figure 3:**
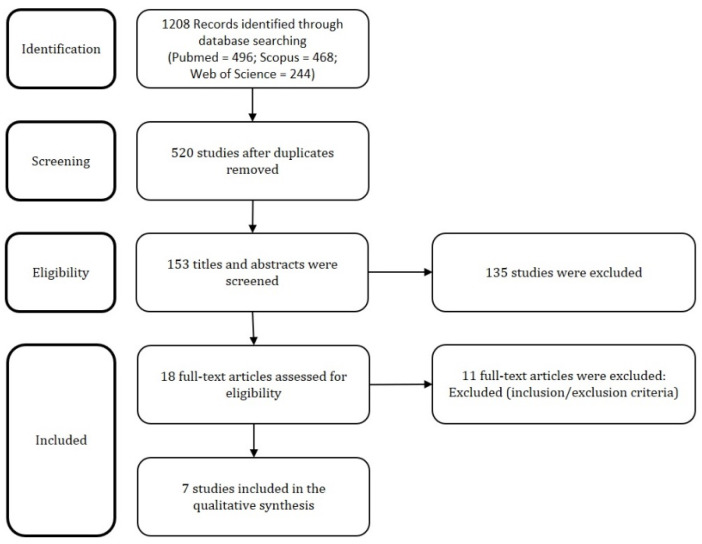
PRISMA (Preferred Reporting Items for Systematic Reviews and Meta-Analyses) flow chart with summary of search results.

The Figure 3 presents the PRISMA flow chart with summary of search results.

We identified 1208 articles on Pubmed, Scopus, and Web of Science. After duplicates exclusion, 153 records underwent first-step screening of titles and abstracts. Eighteen full texts were considered for eligibility, and after reading them, eleven were excluded for being unfit according to the inclusion and exclusion criteria. Finally, seven studies were included in the review, for a total of nine patients diagnosed with EC with IM/diff including the case reported by us.

Table 2 summarizes the clinical and pathological features, treatment modalities, and outcomes of all published cases of primary ECs with IM/diff [9,10,11,13,14,15,16]. Mean and median age were respectively 64 and 62 years (range 49–81 years). Concerning pathological features, a neoplastic precursor was identified only in three of seven cases with available information, and it was represented by AEH (2/7, 28.57%) or by an endometrial polyp with intestinal-type metaplasia (1/7, 14.28%); in the majority of the cases (5/7, 71.42%), it was not identified. Mixed ECs (showing an associated endometrioid component) and pure tumor forms (devoid of endometrioid component) were equally distributed (both 3/6, 50% of the cases). At least one of the main markers of intestinal differentiation (CK20 or CDX2) was expressed in all tested tumors. With the exception of our patient who received neoadjuvant chemotherapy (1/7, 14.28%), the majority underwent up-front surgery (6/7, 85.72%). Three patients (3/7, 42.85%) underwent hysterectomy with bilateral salpingo-oophorectomy; while the remaining (4/7, 57.15%) received additional surgical procedures (pelvic/paraaortic lymphadenectomy, appendectomy, omental biopsy). Radiotherapy was administered to only one patient, while none of the remaining patients received additional adjuvant therapy. Stage disease at presentation was low-stage (IA) in 4/7 patients (57.15%) and high-stage (III–IV) in the remaining 3/7 cases (42.85%). During clinical follow-up, available for five cases, three patients underwent tumor recurrences (3/5, 60%), occurring in the vagina, vulva, and peritoneal cavity. At final follow-up evaluation, two patients were alive with disease (2/5, 40%), 2 patients were alive with no evidence of disease (2/5, 40%), and 1 died of disease (1/5, 20%) after 21 months for peritoneal carcinosis.

## 4. Discussion

IM/diff is an unusual morphological form of mucinous metaplasia that can rarely occur in benign and malignant endometrial lesions [14,17,18]. It has been exceptionally described in normal endometrium, endometrial glandular hyperplasia, and endometrial polyps; little is known about its clinical implications in benign contexts, but reports from the literature suggested follow-up and/or additional clinical investigation. In fact, a patient who underwent removal of an endometrial polyp with IM/diff, finally developed an adenocarcinoma with intestinal-type features [14]. IM/diff can also occur in endometriosis and, interestingly, it has been mainly reported in the appendix and cecum, where the local milieu may favor the metaplastic transformation; alternatively, colonization of endometriosis by normal colic mucosa could be responsible for this process. IM/diff had shown no significant clinical implications in endometriosis; however, pathologists should be aware of this rare condition, since florid forms may mimic primary mucinous tumors, especially in the appendix [19,20].

Malignant endometrial tumors showing mucinous intestinal-type features are extremely rare, and include ECs with IM/diff, primary endometrial yolk-sac tumors with endodermal-intestinal differentiation, and metastases. Before considering a diagnosis of a primary EC with IM/diff, careful pathological work-up should exclude a secondary involvement from cervical, ovarian, gastrointestinal, or pancreatobiliary tumors. When clinical information is unavailable or scarce, a mainly extra-uterine disease with extensive peritoneal spread, diffuse lymphovascular invasion, extrinsic uterine infiltration with minor endometrial involvement, and absence of AEH favors a metastasis from an extra-uterine carcinoma [3,21]. Immunohistochemistry is of limited value since intestinal metaplastic areas are usually negative for hormone receptors and present overlapping immunophenotype with extra-uterine mucinous tumors. HPV molecular testing can help in excluding a secondary involvement by a HPV-related primary cervical tumor [22]; the exclusion of a primary cervical carcinoma could be supported also by the absence of endocervical adenocarcinoma precursors, such as adenocarcinoma in situ (usual-type and gastric-type), lobular endocervical glandular hyperplasia (LEGH), and endometriosis. It should be reminded that the endometrium can be occasionally colonized by metastases (especially from appendix) replacing the epithelium of endometrial glands with neoplastic goblet cells [23].

ECs exhibiting intestinal-type features share morphological and phenotypical features with pure forms or somatic-type variants of yolk-sac tumors, in particular from those cases presenting endodermal-intestinal differentiation with glandular architecture and expression of CDX2 and CK20. Distinctive cytological features (i.e., apical and subnuclear cytoplasmic clearing) and the expression of germ-cell markers (SALL-4, Glypican-3 and AFP) help in the recognition of this entity [24].

Finally, ECs exhibiting IM/diff should be distinguished from other types of mucinous differentiation occurring in ECs. Usual-variants of mucinous carcinomas of the endometrium frequently present, at least focally, endocervical-like glands; in addition, they typically express hormonal receptors (ER and PR) [1,5]. Endometrial carcinomas exhibiting gastric-type differentiation had been recently elucidated by Wong et al. colleagues. They present overlapping morphological and clinical features (aggressive behavior) with gastric-type adenocarcinoma of the cervix and vagina; interestingly, one of the reported cases by the authors showed benign gastric-type mucinous glands in the background endometrium, reminiscent of in situ and lobular endocervical glandular hyperplasia (LEGH) and gastric-type vaginal adenosis [8]. In the case reported by us, the intestinal component of tumor showed intestinal-type morphology, while gastric-type tumor-differentiation and benign endometrial lesions with gastric-type metaplasia were not found. In addition, according to Wong et colleagues, a typical endometrioid component should be absent by definition in endometrial gastric (gastro-intestinal)-type adenocarcinomas, while it was present in in our case [8].

Here, we present a peculiar case of EC with mixed endometrioid and mucinous features in the form of IM/diff, characterized by MMR defects (MSH2/MSH6 loss of expression and MSI-H); the abovementioned differential diagnoses (metastases, and yolk-sac tumor with intestinal-like features, other variants of mucinous endometrial carcinomas) were excluded after wide clinical, morphological and immunohistochemical work-up. Primary upfront surgery, as well as palliative surgery, were not considered in view of the likely complexity attended, mimicking ovarian cancer carcinomatosis at the liver site [25,26,27].

We performed a systematic review of the English Literature in multiple databases and identified only eight previous cases of EC with IM/diff, arising in patients of 49 to 81 years of age (Table 2) [9,10,11,13,14,15,16]. IM/diff can manifest in ECs with a wide spectrum of morphological features, ranging from the presence of sole goblet cells to the association of goblet cells with additional gastrointestinal-type cells, such as enterocyte-like cells, neuroendocrine cells, and Paneth-like cells. Histological presentation similar to colic mucinous adenocarcinoma [9] or as pseudo-myxomatous lakes containing strips of atypical glands can also happen [14]. Besides intestinal-type features, most of the reported tumors showed immunohistochemical positivity for gastrointestinal markers (CK20, CK7, CDX2, villin, MUC6, and/or MUC2); PAX8, ER, and PR were usually negative, focally/weakly expressed or with positivity confined in tumor components lacking IM/diff features.

We could distinguish two variants of ECs with IM/diff, as to the presence (mixed forms) or absence (pure forms) of an endometrioid component, that appear to be equally distributed. A different pathogenesis may be supposed for pure and mixed forms. At least in some mixed forms, the metaplastic process could represent a relatively late event in the step-like transformation of type I endometrial carcinomas, and AEH could represent the precursor lesion. AEH was detected in the background endometrium of two cases: one a mixed form, while it was unclear if an endometrioid component was present in the second case. In both patients, no IM/diff was found in benign glands [11,13]. Alternative pathogenic mechanisms and precursors beside AEH may be considered for pure forms: the tumor reported by Rubio arose after previous removal of an endometrial polyp with IM/diff, and AEH was not found in the background endometrium [14]. In our review, a precursor lesion was not found or reported in the majority of the cases: partial sampling or extensive tumor growth (destroying non-invasive areas) can be involved in the failure to recognize precursors of mixed or pure forms, as well as endometrioid components. Further studies are required.

In fact, EC with IM/diff could represent a more aggressive variant in comparison to endometrioid or mucinous (non-intestinal type) ECs. Evaluation of clinical features points out that even if the majority of the patients were diagnosed with stage I disease, a significant fraction presented at advanced stage (stage III or IV), including our case. Furthermore, follow-up data (available for five patients) showed three disease recurrences; interestingly, two of them occurred in patients presenting with stage IA disease [9,13], including one carcinoma leading to patient’s death after 21 months of follow-up. However, the extent of IM/diff areas required to increase the aggressiveness of an EC is unknown; in the majority of papers, it was unclear if IM/diff features were focal or diffuse. With the exception of case described by Mogor in which the recurrent tumor exhibited intestinal differentiation, in the remaining cases, including our one, histological evaluation of the metastatic/recurrent tumor was not performed, and for this reason, it is not clear if the intestinal component is more aggressive in comparison to the endometrioid one [13].

Besides the wide spectrum of morphological presentation, ECs were classified by the TCGA into four main genomic subgroups [28]. The ProMise (Proactive Molecular Risk Classifier for Endometrial Cancer) algorithm, a surrogate of the molecular TCGA classification, allows the individuation of analogous (but not identical) subcategories with easier techniques [29]. One of those classes is represented by tumors with MSI-H, characterized by MLH1-promoter hypermethylation or mutations of MMR genes (MLH1, MSH2, MSH6, and/or PMS2). In our case, the concomitant loss of MSH2 and MSH6 proteins expression in both endometrioid and intestinal tumor components provided indirect evidence of a shared molecular alteration and a very-likely clonal origin from a common neoplastic progenitor. Moreover, despite not finding AEH or intestinal metaplasia in the background endometrium, we identified the same MMR-defects of the EC in normal-appearing endometrial glands. This finding usually occurs as an early event in patients with Lynch Syndrome, due to germline mutations of MMR-genes [30]. Lynch Syndrome was highly suspected for our patient in consideration of molecular findings as well as family history; genetic counseling was warranted but not yet performed.

As reported by different authors, ECs with MMR-defects can reveal peculiar gross and microscopic features: even if they mostly exhibit endometrioid-differentiation, rarer morphological subtypes had been described, including undifferentiated, dedifferentiated, mixed-type or clear cell carcinomas, and carcinosarcomas [31,32]. Only a previous case of EC with IM/diff and MMR-defects was described; the tumor resembled a colic mucinous adenocarcinoma without evidence of an endometrioid component and showed MLH1 promotor hypermethylation and MSI-H [9]. In our review, our case represents the first report of a mixed endometrioid and mucinous-intestinal type EC showing MMR-defects, including immunohistochemical loss of MSH2/MSH6 and MSI-H. These cases highlight an additional unusual morphological differentiation that can be detected in the context of ECs with MSI-H/MMR defects.

The neoadjuvant chemotherapy (NACT)-induced morphological changes on ovarian carcinoma are well characterized, and they do not include metaplastic changes in the form of intestinal-type differentiation [33]; those occurring in endometrial carcinomas have been poorly investigated [34] since most of the studies focused on the effects of the hormonal therapy [33]. Even if we could not exclude the occurrence of a “possible” NACT-induced metaplastic change (with intestinal-type features) in our case, the fact that none of the remaining patients described in the review underwent NACT supports the hypothesis that (at least for those cases) IM/diff can represent an original and unusual morphological presentation of naïve EC.

In conclusion, IM/diff is an unusual phenomenon that can occur in endometrial carcinomas; besides the detection of goblet cells, additional morphological criteria are needed to better define this entity. Similarly to ECs with gastric-type differentiation, there is suggesting evidence that ECs showing IM/diff could represent a more aggressive variant because of relatively frequent high-stage disease presentation and disease recurrences. In addition, patients with this uncommon variant may be screened for MSI/MMR-defects and Lynch Syndrome.

## Figures and Tables

**Table 1:** Results of immunohistochemical analysis with separate evaluation for endometrioid and mucinous-intestinal tumor components.

E+/M+	E+/M−	E−/M+	E-/M-
CK7, *p*53 (wild-type pattern), MSH1, PMS2, synaptophysin (<5%), chromogranin (<5%), *p*16 (patchy)	PAX8, ER, PR	CDX2, CK20	PAX2, PTEN, MSH2, MSH6, ARID1A, SALL4, AFP, Glypican-3

E: endometrioid component; M: mucinous-intestinal component; +: positive immunostaining; **−**: negative immunostaining.

**Table 2:** Literature review of endometrial carcinomas showing intestinal type metaplasia/differentiation.

Authors	Age (years)	Original Diagnosis	Precursor	E-comp	Features of IM/diff	IHC/MA	Treatment	FIGO Stage	FU
Ardighieri et al., 2020	49	G2 mixed endometrioid and mucinous-intestinal type differentiated carcinoma	NI (*)	yes (G2)	TCC, GC	Intestinal component (§): Positive: CDX2, CK20, CK7, MLH1, PMS2, p16 (patchy) Negative: PAX8, ER, PR, MSH2, MSH6 MA: HG-MSI	NCHT+H+ BSO+PLND	IVB	AWD at 6 months
Mogor et al., 2019 [13]	58	G2 intestinal-like mucinous adenocarcinoma (tumor recurrence)	AEH	unclear	GC	Positive: CK7, CDX2, CK20 Negative: ER, PR, PAX-8	H + BSO + PLND	IA	Vaginal and vulvar recurrences. NED at 87 months
Trippel et al., 2017 [9]	62	Intestinal differentiated mucinous adenocarcinoma	NI	no	Solid/cribriform patterns; abundant extracellular mucin (°,$)	Positive: CDX2, CK7, CK20, p16 (scattered), MSH2, MSH6 Negative: PAX8, WT1, ER, PR, synaptophysin, chromogranin, vimentin, AFP, SALL4, Glypican3, MLH1, PMS2. MA: HG-MSI; MLH1 promoter hypermethylation	H + BSO + PLND	IA	Peritoneal recurrence. DOD at 21 months
Rubio et al., 2016 (case 1) [14]	81	Mucinous adenocarcinoma of gastrointestinal type	Previous endometrial polyp with IM/diff	no	Pseudomyxomatous mucinous lakes; atypical glands; columnar cells with mucin-laden cytoplasm ($)	Positive: CK7, CK20, CDX2, villin, MUC2, MUC5AC, MUC6, p16 (patchy). Negative: vimentin, PAX8, ER, PR	H + BSO	IIIA	NA
Buell-Gutbrod et al., 2013 [11]	55	G1 endometrioid adenocarcinoma with endocervical and intestinal-type mucinous differentiation	AEH	yes (G1)	GC	Intestinal areas: Positive: CK7, CDX2, CEA Negative: synaptophysin, chromogranin, CK20	H + BSO	IA	NA
Nieuwenhuizen et al., 2007 (case 10) [15]	NA	G2 endometrial adenocarcinoma with goblet cells metaplasia	NA	NA	GC	NA	NA	NA	NA
Nieuwenhuizen et al., 2007 (case 11) [15]	NA	G2 endometrial adenocarcinoma with goblet cells metaplasia	NA	NA	GC	NA	NA	NA	NA
Zheng et al., 1995 [16]	71	Mucinous adenocarcinoma with intestinal differentiation	NI	no	TCC, NC, GC	Positive: CEA, synaptophysin, chromogranin, ER, PR, NSE	H + BSO + PLND + PALD + OB + AP + PRT	IIIC	Peritoneal recurrence. AWD at 14 months
Berger et al., 1984 [10]	72	G1 endometrial carcinoma of intestinal type	NI	yes (G1)	TCC, SRC, GC, PC, NC	Positive: gastrin, CKK, somatostatin, lysozyme, CEA	H + BSO	IA	NED

(*): Focal loss of MSH2 and MSH6 in normal-appearing endometrial glands; (§): further details in Table 1; (°): mimicking mucinous adenocarcinoma of the colon; ($): cell types were not accurately reported. E-comp: endometrioid component; IM/diff: intestinal metaplasia/differentiation; IHC/MA: immunohistochemical and molecular analysis; FU: follow-up; NI: not identified; TCC; tall columnar cells similar to enterocyte-like cells; GC: goblet cells; HG-MSI: high-grade microsatellite instability; NCHT: neoadjuvant chemotherapy; H: hysterectomy; BSO: bilateral salpingo-oophorectomy; PLND: pelvic lymph node dissection; AWD: alive with disease; AEH: atypical endometrial hyperplasia; NED: no evidence of disease; DOD: dead of disease; NA: not assessed; NC: neuroendocrine cells; PALD: para-aortic lymphadenectomy; OB: omental biopsy; AP: appendectomy; PRT: pelvic radiation therapy; SRC: signet ring cells; PC: Paneth cells.
